# Flavonoid Synthesis Pathway Response to Low-Temperature Stress in a Desert Medicinal Plant, *Agriophyllum Squarrosum* (Sandrice)

**DOI:** 10.3390/genes15091228

**Published:** 2024-09-20

**Authors:** Pengshu Zhao, Xia Yan, Chaoju Qian, Guorong Ma, Xingke Fan, Xiaoyue Yin, Yuqiu Liao, Tingzhou Fang, Shanshan Zhou, Ibrahim Awuku, Xiao-Fei Ma

**Affiliations:** 1 Key Laboratory of Ecological Safety and Sustainable Development in Arid Lands, Northwest Institute of Eco-Environment and Resources, Chinese Academy of Sciences, Lanzhou 730000, China; 2 Key Laboratory of Stress Physiology and Ecology in Cold and Arid Regions of Gansu Province, Northwest Institute of Eco-Environment and Resources, Chinese Academy of Sciences, Lanzhou 730000, China; 3 University of Chinese Academy of Sciences, Beijing 100049, China; 4 Key Laboratory of Inland River Ecohydrology, Cold and Arid Regions Environmental and Engineering Research, Northwest Institute of Eco-Environment and Resources, Chinese Academy of Sciences, Lanzhou 730000, China; 5 Gulang County Sand Prevention and Control Technology Promotion Center, Wuwei 733100, China

**Keywords:** *Agriophyllum squarrosum*, cold tolerance, flavonoids, medicinal plant, naringenin biosynthesis

## Abstract

**Background/Objectives:** *Agriophyllum squarrosum* (L.) Moq. (*A. squarrosum*), also known as sandrice, is an important medicinal plant widely distributed in dunes across all the deserts of China. Common garden trials have shown content variations in flavonoids among the ecotypes of sandrice, which correlated with temperature heterogeneity in situ. However, there have not been any environmental control experiments to further elucidate whether the accumulation of flavonoids was triggered by cold stress; **Methods:** This study conducted a four-day ambient 4 °C low-temperature treatment on three ecotypes along with an in situ annual mean temperature gradient (Dulan (DL), Aerxiang (AEX), and Dengkou (DK)); **Results:** Target metabolomics showed that 12 out of 14 flavonoids in sandrice were driven by cold stress. Among them, several flavonoids were significantly up-regulated, such as naringenin and naringenin chalcone in all three ecotypes; isorhamnetin, quercetin, dihydroquercetin, and kaempferol in DL and AEX; and astragalin in DK. They were accompanied by 19 structural genes of flavonoid synthesis and 33 transcription factors were markedly triggered by cold stress in sandrice. The upstream genes, *AsqAEX006535*−*CHS*, *AsqAEX016074*−*C4H*, and *AsqAEX004011*−*4CL*, were highly correlated with the enrichment of naringenin, which could be fine-tuned by *AsqAEX015868*−*bHLH62*, *AsqAEX001711*−*MYB12,* and *AsqAEX002220*−*MYB1R1*; **Conclusions:** This study sheds light on how desert plants like sandrice adapt to cold stress by relying on a unique flavonoid biosynthesis mechanism that regulating the accumulation of naringenin. It also supports the precise development of sandrice for the medicinal industry. Specifically, quercetin and isorhamnetin should be targeted for development in DL and AEX, while astragalin should be precisely developed in DK.

## 1. Introduction

Terrestrial plants survive diverse stresses, including droughts, low temperatures, salinity, UV-B, etc., and they face more challenges due to frequent abnormal climate changes [1,2,3,4,5,6]. To defend and endure the stressful environments, they can produce plenty of protective phytochemicals, such as polyphenolic compounds, saponins, monoterpenes, and phytates [7,8,9,10,11]. This is a universal phenomenon that plants produce complex secondary metabolite mixtures in response to various stresses, exhibiting the phytochemical diversity [12]. As the most famous and abundant protective phytochemicals, flavonoids perform multiple physiological functions in plant growth and in response to various stresses, including regulating plant hormone signals, antioxidation, scavenging free radicals, and participating in vital life processes such as photosynthesis, morphogenesis, and chemical signaling [13,14,15].

In addition, flavonoids are the largest class of polyphenols discovered to date, and are estimated to encompass over 8000 metabolites with a common diphenylpropane A skeleton structure (C6-C3-C6) [16]. This structure consists of two aromatic rings connected by a three-carbon chain and is classified into seven subclasses based on the degree of oxidation of the central ring: flavanols, anthocyanins, flavanones, flavonols, isoflavones, flavones, and chalcones [17]. The biosynthesis of flavonoids in plants occurs at the intersection of the acetic acid and shikimic acid pathways (polyketide pathway), which initiates from the general phenylpropane pathway (GPP). Subsequently, a series of consecutive enzymes, including phenylalanine ammonia lyase (PAL), cinnamate-4-hydroxylase (C4H), 4-coumarate-CoA ligase (4CL), and chalcone synthase (CHS), synthesize various stable flavonoids [17]. Previous research has indicated that the levels of hormones within plants can be influenced by various stressors, thereby leading to the expression of hormone-related regulatory genes, and regulating the important genes of the biosynthetic pathway of the flavonoids [15,18,19]. The MYB–bHLH–WDR (MBW) tertiary complex was widely identified, which is composed of three transcription factor families (MYB, bHLH, and WD40) that regulate flavonoid synthesis-related genes [20,21,22,23]. Research on the flowers of *Pyrus hopeiensis* demonstrated that *bHLH* and *MYB* regulate the expression of genes associated with flavonoid synthesis, thereby influencing flavonoid biosynthesis and responding to low-temperature stress [24]. The study of *Tetrastigma hemsleyanum* seedlings revealed that the expression of *PAL*, *CL*, *CHS*, *ANR*, *FLS*, and *LAR* was up-regulated under cold stress, resulting in a significant increase in key flavonols such as catechins, epicatechins, rutin, and quercetin [25]. In *Fagopyrum tataricum* (*F. tataricum*) hairy roots, the biosynthesis of rutin was directly repressed by JA-responsive TFs, such as *FtMYB13*, *FtMYB14*, *FtMYB15*, and *FtMYB16* [26], and rutin synthesis in the sprouts could be up-regulated to a higher-level under cold stress [27]. In tobacco plants, overexpression of *CHS* could induce flavonoid accumulation in tobacco leaves (rutin, quercetin, kaempferol-3-rutinoside, and kaempferol-glucopyranoside), and improve drought resistance [28]. Research on *Camellia sinensis* has found that UV-B could induce the activation of *MYB12* which, when combined with the promoters of flavonoid biosynthesis genes (*CsFLS*, *CsLAR*, and *CsDFR*), leads to changes in flavonoid content in the tea leaves [29]. In a word, the biosynthesis of flavonoids was largely triggered across different economic plant species under various stresses, with tissue- and species-specific regulations.

As desert plants survive prolonged exposure to multiple stressful environments, they could evolve specific regulation mechanisms in flavonoid biosynthesis. To date, however, most studies still primarily focus on the effects of drought and salt stress on the flavonoid biosynthesis of certain medicinal and edible desert plants like *Hippophae rhamnoides* (*H. rhamnoides*) and *Lycium barbarum* (*L. barbarum*) [30,31,32,33,34,35]. Few have addressed how flavonoid synthesis in desert plants responds to cold stress, which commonly and frequently happens in deserts. *Agriophyllum squarrosum* (L.) Moq. (*A. squarrosum*), commonly known as sandrice, is an annual medicinal desert plant from the Chenopodioideae subfamily, and is widely distributed in areas with an annual mean temperature (AMT) of 2~10 °C [36]. Due to its highly heterogeneous growth environment, it produces a large number of secondary metabolites, such as flavonoids, organic acids, terpenoids, and alkaloids [37]. Among them, the content of flavonoids is abundant, including rutin, isoquercitrin, quercetin, and isorhamnetin, etc. [36,38]. Common garden trials have indicated that variations in the content of flavonoids among ecotypes of sandrice was related to the hydrothermal heterogeneity in situ [36,38]. However, more controlled experiments are still needed to verify how flavonoid biosynthesis in sandrice responds to low-temperature stress.

In this study, we hypothesized that cold stress might trigger the accumulation of flavonoids in sandrice. To test our hypotheses, based on flavonoid targeted metabolomics and transcriptomic analyses, this study conducted a four-day ambient 4 °C low-temperature treatment experiment with three ecotypes from different AMT in situ gradients, DL (Dulan), AEX (Aerxiang), and DK (Dengkou). By discussing the phytochemical diversity and genetic diversity among different ecotypes, we aimed to supply a new example to better understand how a medicinal plant adapts to the environmental heterogeneity through flavonoid biosynthesis. Our findings will deepen the understanding of the metabolic processes of sandrice in response to low-temperature stress, and provide a theoretical basis for further precise utilization and development of flavonoids.

## 2. Materials and Methods

### 2.1. Plant Growth, Cold Treatment, and Sample Collection

The seeds of three natural ecotypes of sandrice, DL, AEX, and DK, were collected from the original habitat in 2014, and the data of the environmental variables in situ were downloaded from WorldClim (Table 1). Their AMTs are 3.4 °C, 6.38 °C, and 8.31 °C, respectively. Seeds were grinded with 1 mm diameter of sand (the ratio of sand to seed was 2:1, *v*/*v*) to break dormancy and ensure the uniform germination of the wild sandrice seeds from the three ecotypes [39], and then were germinated overnight in a dark incubator at 25 °C. The next day, the germinated seeds were sown into pots filled with sand and nutrient soil (9:1, *v*/*v*). Once the cotyledons grew, the pots were placed under artificially simulated climatic conditions: with a photoperiod of 16 h/8 h (light/dark) with approximately 30–50% relative humidity (Rh) and temperature of 30 °C/25 °C (light/dark) in the greenhouse at the Northwest Institute of Eco-Environment and Resources of the Chinese Academy of Sciences. The synthetic light source was composed of one LED red and blue filling light tube (WEGA/Guixiang, 90W No. 1 spectrum) and two LED full-spectrum synthetic succulent filling light lamps (WEGA/Guixiang, 36 W synthetic succulent spectrum). There was only one healthy plant with the same growing consistency maintained in each pot, this was ensured by removing extra plants. A total of 36 pots were divided into 6 groups (6 pots for each group), including the 3 control groups (CDL, CA, and CDK) and 3 cold-treatment groups (CCDL, CCA, and CCDK). Until 40 days after germination, based on a systematic study of sandrice development stages [40], sandrice is in its vigorous period. At this time, the three ecotypes of sandrice exhibited morphological differences, while the morphological structures of every ecotype remained relatively stable, and cold treatment began. The plants in the control group were continuously cultured at 30 °C/25 °C (light/dark), while the plants in the cold-treatment group were exposed to 8 °C/4 °C (light/dark). After 4 days of treatment, a slight leaf whitening with eyesight was observed, at which point the experiment concluded and the above-ground tissue samples were randomly pooled from 6 plants in each group, then were immediately frozen in liquid nitrogen, and stored at −80 °C in a refrigerator. A total of 6 samples with 3 biological replicates of each treatment were analyzed. Throughout the experiment, all plants were timely watered to maintain a suitable soil moisture content (20%–25%). The soil moisture content was monitored using a soil moisture sensor (Shun Koda TR-6, Beijing, China) at 9:00 AM.

### 2.2. Plant Physiological and Morphological Measurements

The Leaf relative water content (Leaf RWC, %), actual photochemical quantum yield (Fv’/Fm’), actual photosynthetic efficiency or quantum yield of photosystem II (Phi2), unregulated energy dissipation (PhiNO), non-photochemical quenching (PhiNPQ), above-ground biomass (AGB), Plant Height, leaf area (LA), stem diameter (SD), and basal branch long (BBL) were utilized as parameters to assess the physiological and morphological differences related to low-temperature resistance among the three ecotypes of sandrice. Fv’/Fm’ (FvP/FmP) represents the actual photochemical quantum yield, while Fv/Fm denotes the maximum quantum yield of photosystem II. Phi2, which is directly related to Fv/Fm, can be calculated using the equation: Φ2 = (Fm’ − Fs’)/Fm’, where Fs’ represents the steady-state fluorescent yield of light-adapted leaves. PhiNPQ can also be calculated using the equation: ΦNPQ = (Fm − Fm’)/Fm’. In this context, Φ represents Phi. PhiNO can be quantified using the equation: 1 − (Φ2 + ΦNPQ). This is because the sum of Phi2, PhiNPQ, and PhiNO must equal 1. Fresh weight (FW), turgid weight (TW), and dry weight (DW) of four leaves from each sample were immediately measured after isolating them from the plants. The leaves were then rehydrated in distilled water at 4 °C for 24 h until fully turgid, and subsequently dried at 70 °C for 72 h until a constant mass was attained. Leaf RWC was calculated using the following formula: Leaf RWC (%) = (FW − DW)/(TW − DW) × 100 [41]. Measurements of Fv’/Fm’, Phi2, PhiNO, and PhiNPQ were performed using MultispeQ (MultispeQ V2.0, PhotosynQ LLC Co. Ltd., East Lansing, MI, USA). The SD of the plants was measured using a digital vernier caliper (DL91150, Deli Co. Ltd., Yuyao, China). The AGB was weighed using a 1/10,000 electronic analytical balance. Plant Height and BBL were measured with a ruler. Physiological data with six biological replicates for each treatment were statistically analyzed using a one-way analysis of variance (ANOVA) and t-tests at the 0.05 and 0.01 significance levels using IBM SPSS 17.0 Statistics (SPSS Inc., Armonk, NY, USA). The data with replicates were presented as the mean of the six replicates, with significance markers. The bar chart was generated using GraphPad Prism 8.2.0 software.

### 2.3. Metabolite Extraction and Analysis

Approximately 100 mg of samples were extracted with 2 mL in 70% *v*/*v* aqueous methanol, using ultrasonic waves for 30 min at a time, for a total of 2 h, and the mixed extracts were concentrated to nearly dry on a rotary evaporator at 35 °C under reduced pressure. Before analysis, the residue was dissolved in 200 μL of 50% methanol/water and transferred to insert-equipped vials.

The sample extracts were analyzed using the UPLC–Orbitrap–MS system (UPLC, Vanquish; MS, QE). The analytical conditions were as follows: UPLC column, Waters HSS T3 (50*×2.1 mm, 1.8 μm); column temperature, 40 °C; flow rate, 0.3 mL/min; injection volume, 2 μL; solvent system, water (0.1% acetic acid); acetonitrile (0.1% acetic acid); and gradient program, 90:10 *v*/*v* at 0 min, 90:10 *v*/*v* at 2.0 min, 40:60 *v*/*v* at 6.0 min, 40:60 *v*/*v* at 8.0 min, 90:10 *v*/*v* at 8.1 min, and 90:10 *v*/*v* at 12.0 min.

HRMS data were recorded on a Q Exactive hybrid Q-Orbitrap mass spectrometer equipped with a heated ESI source (Thermo Fisher Scientific, Waltham, MA, USA), utilizing the SIM MS acquisition methods. The ESI source parameters were set as follows: spray voltage, −2.8 kV; sheath gas pressure, 40 arb; aux gas pressure, 10 arb; sweep gas pressure, 0 arb; capillary temperature, 320 °C; and aux gas heater temperature, 350 °C.

Data were acquired on the Q-Exactive using the Xcalibur 4.1 (Thermo Scientific, Waltham, MA, USA), and processed using the TraceFinder™4.1 Clinical (Thermo Scientific). Quantified data were output into excel format. Principal component analysis (PCA) and orthogonal partial least squares discriminant analysis (OPLS-DA) were performed using the Metware Cloud, a free online platform for data analysis (https://cloud.metware.cn, accessed on 27 February 2024). The differentially accumulated flavonoids (DAFs) were filtered by variable importance in the projection (VIP) ≥ 1 and *p* ≤ 0.05. Venn and hierarchical cluster heatmap analyses were performed using the OECloud tools at https://cloud.oebiotech.com, accessed on 24 November 2023.

### 2.4. RNA Isolation and Library Preparation

Total RNA was extracted from 18 sandrice above-ground tissue samples using the TRIzol reagent (Invitrogen, Carlsbad, CA, USA), according to the manufacturer’s protocol. RNA purity and quantification were evaluated using the NanoDrop 2000 spectrophotometer (Thermo Scientific, USA). RNA integrity was assessed using the Agilent 2100 Bioanalyzer (Agilent Technologies, Santa Clara, CA, USA). Then, the libraries were constructed using VAHTS Universal V6 RNA-seq Library Prep Kit according to the manufacturer’s instructions. The transcriptome sequencing and analysis were conducted by OE Biotech Co., Ltd. (Shanghai, China).

### 2.5. RNA Sequencing and Differentially Expressed Genes Analysis

The libraries were sequenced on an Illumina Novaseq 6000 platform, generating 150 bp paired-end reads. The raw sequencing files of the transcriptomic data are now available in the NCBI SRA database under the accession number PRJNA1088300. The raw reads in fastq format were initially processed using fastp, and low-quality reads were removed. The resulting clean reads were then mapped onto the AEX genome (data unpublished) using HISAT2. The expression levels of each gene were represented by the FPKM value, which was calculated and obtained by an HTSeq-count using the corresponding read counts. Differential expression analysis was performed using DESeq2, with a threshold of *q*-value < 0.05 and |log_2_ (fold change)| ≥ 1 set for significantly differential expression genes (DEGs). To recognize the biological role and function of DEGs, KEGGs (Kyoto Encyclopedia of Genes and Genomes) enrichment analyses were employed. The OECloud tools at https://cloud.oebiotech.com, accessed on 19 July 2024, were used to produce a KEGG enrichment analysis bubble map.

### 2.6. Conjoint Analysis Method

A network diagram was generated using Cytoscape 3.9.1, and its Pearson correlation coefficient (|PCC| ≥ 0.8), and the significance of difference at *p* < 0.05 was calculated and screened by SPSS 17.0 Statistics. The KEGG pathway heatmap was created based on the upstream and downstream relationships of flavonoid synthesis enzymes and metabolites in the KEGG Pathway Database (https://www.kegg.jp/kegg/pathway.html, accessed on 24 November 2023).

### 2.7. Q-PCR Analysis

RNA extraction in CDL, CCDL, CA, CCA, CDK, and CCDK, and reverse transcription was respectively obtained using the RNAprep Pure Plant Plus Kit (Polysaccharides & Polyphenolics-rich) and FastKing gDNA Dispelling RT SuperMix (TIANGEN Biochemical Technology Co., Ltd., Beijing, China). Q-PCR primers were designed using Primer Premier 5 software (Premier Biosoft, Palo Alto, CA, USA) (Table S1). *AsUBC22* and *AsPP2A*, with mean values based on previous laboratory studies, were used as internal reference genes [42]. Target gene amplification was performed using the SuperReal PreMix Plus (SYBR Green) of TIANGEN and a Real-Time Q-PCR System (Mx3000P, Agilent Technologies Co., Ltd., Santa Clara, CA, USA). Three technical replicates were performed for each biological replicate. Finally, a normalized expression analysis was conducted for each sample using 2^−ΔΔCt^ to verify the reliability of the transcriptome data.

## 3. Results

### 3.1. Morphological and Physiological Responses to Low-Temperature Stress in Sandrice

**Figure 1:**
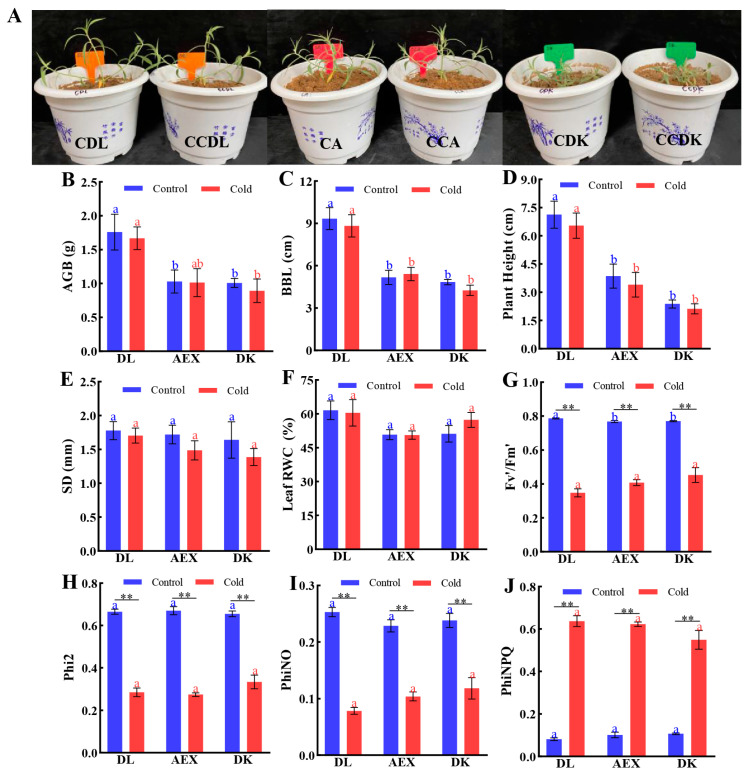
(**A**) Phenotype of DL, AEX, and DK after cold stress. CDL, CA, and CDK represent the control of DL, AEX, and DK, respectively. CCDL, CCA, and CCDK represent the cold stress of DL, AEX, and DK, respectively; (**B**) AGB (above-ground biomass, g); (**C**) BBL (basal branch long, cm); (**D**) Plant Height (cm); (**E**) SD (stem diameter, mm); (**F**) Leaf RWC (leaf relative water content, %); (**G**) Fv’/Fm’; (**H**) Phi2; (**I**) PhiNO; (**J**) PhiNPQ. The bars indicate mean values ± SE (*n* = 6). Within the same treatment (control or cold), the same superscripts indicate no significant difference (*p* > 0.05), different superscripts indicate a significant difference (*p* < 0.05); asterisks on shoulder lines indicate statistically significant differences between control treatment and cold-stress treatment of each ecotype: **, *p* < 0.01.

Throughout the experiment, the soil moisture content in both the control group and the low-temperature treatment group was maintained at approximately 25%. Although some growth parameters, such as AGB, BBL, and Plant Height, varied among the three ecotypes, the four-day low-temperature treatment did not induce any significant morphological changes within all three ecotypes compared to the control (Figure 1A–E). However, significant differences were observed in the photosynthetic parameters between the low-temperature treatment group and the control group within each ecotype. Specifically, Fv’/Fm’ (Figure 1G), Phi2 (Figure 1H), and PhiNO (Figure 1I) significantly decreased by 56%–69%, 47%–59%, and 41%–50%, respectively, while PhiNPQ (Figure 1J) significantly increased by 5–8 times. These phenomena suggested that under cold stress the leaves of sandrice were subject to photoinhibition, with a reduced capacity for photosynthetic electron transport and a decline in photosynthetic rates.

### 3.2. Flavonoids Variations of Sandrice under Low-Temperature Stress

**Figure 2:**
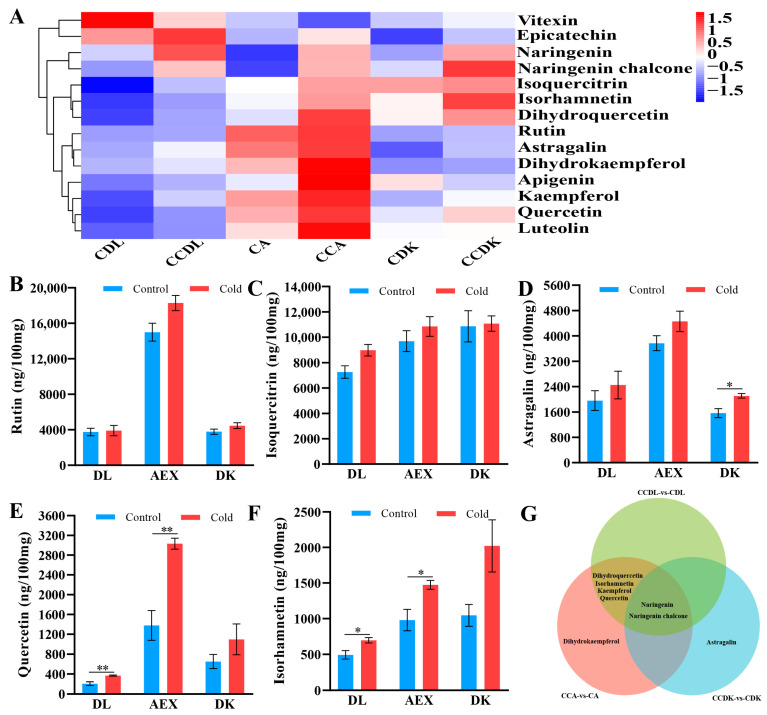
The flavonoid-targeting metabolites identified in the above-ground tissue of sandrice. (**A**) The hierarchical cluster heatmap analysis of metabolites in six groups; (**B**–**F**) 5 flavonoid metabolites with high content; (**G**) Venn diagram of metabolite significance analysis in three ecotypes. *, VIP ≥ 1 and *p* ≤ 0.05; **, VIP ≥ 1 and *p* ≤ 0.01.

The composition and contents of 14 flavonoid metabolites in the above-ground tissue of sandrice are detailed in Table S2, with concentrations ranging from 0.049 to 18,284.928 ng/100 mg. Among them, rutin was the most abundant metabolite in AEX, whereas isoquercitrin predominated in DK and DL. The PCA plot of flavonoid metabolites (Figure S1A) explains the 52.9% (PC1) and 17.5% (PC2) variation, with each treatment’s three replicates falling within the 95% confidence interval. The scatter plot of the OPLS-DA model further supported the significant distinction between samples from the control group and the low-temperature treatment group of the three ecotypes (Figure S1B–D). Notably, the low-temperature treatment groups and control groups for each ecotype were clearly separated, indicating that low-temperature stress drove the biosynthesis of flavonoids in sandrice. The hierarchical clustering heatmap revealed that 12 flavonoid metabolites were up-regulated under low-temperature conditions (Figure 2A). In all treatment groups, rutin (Figure 2B), isoquercitrin (Figure 2C), astragalin (Figure 2D), quercetin (Figure 2E), and isorhamnetin (Figure 2F) were the predominant flavonoids, collectively comprising over 99% of the total flavonoid content. Applying the DAFs selection criteria (VIP ≥ 1 and *p* ≤ 0.05) (Table S3), a total of eight DAFs were identified (Figure 2G). These included two common DAFs (naringenin and naringenin chalcone) shared by all three ecotypes, four DAFs (dihydroquercetin, isorhamnetin, kaempferol, and quercetin) shared by DL and AEX, and a unique differential metabolite (astragalin) in DK and (dihydrokaempferol) in AEX.

### 3.3. DEGs Responding to Low-Temperature Stress in Sandrice

**Figure 3:**
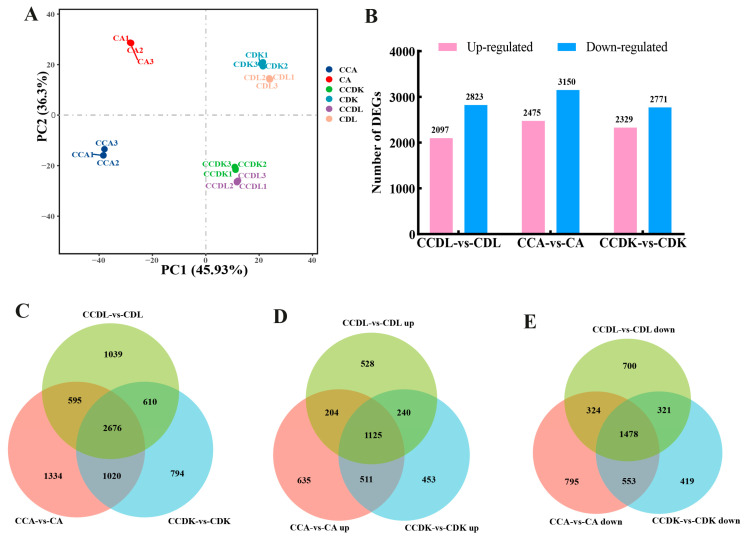
Overall description of DEGs in sandrice in response to low-temperature stress. (**A**) The PCA plot; (**B**) the bar plot of DEGs; (**C**) Venn diagram of all DEGs in the three ecotypes; (**D**) Venn diagram of up-regulated DEGs in the three ecotypes; (**E**) Venn diagram of down-regulated DEGs in the three ecotypes.

A total of 18 samples generated 127.18 G of raw data, resulting in 121.60 G of clean data after the removal of the low-quality reads. The genome mapping rates for each sample ranged from 96.4% to 98.57%, while the unique mapping rates for clean reads varied between 90.53% and 95.28%. The distribution of Q30 bases ranged from 92.63% to 93.17%, and the average GC content was 43.83% (Table S4). In the PCA plot, the 18 samples were divided into control groups and low-temperature treatment groups alongside PC1, while the *Central* lineage (DL and DK), and the *Eastern* lineage (AEX) were alongside PC2 (Figure 3A). These findings indicate that the low-temperature treatment significantly affected all three ecotypes of sandrice at the transcriptome level. Furthermore, it was noticed that the transcriptomic samples of DK and DL clustered together under both the control and low-temperature conditions, suggesting that these two ecotypes may exhibit similar transcriptional responses to cold stress.

**Figure 4:**
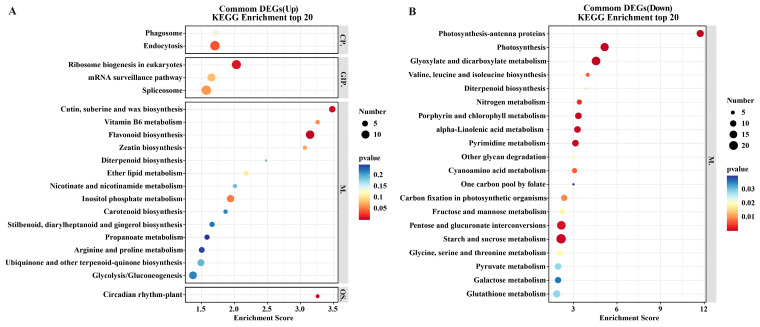
KEGG enrichment analysis bubble map of the three ecotypes of sandrice. (**A**) The bubble map of common up-regulated 1125 DEGs; (**B**) the bubble map of common down-regulated 1478 DEGs. CP., GIP., M., and OS. represent Cellular Processes, Genetic Information Processing, Metabolism, and Organismal Systems, respectively. Dot size represents the number of DEGs, and dot color reflects the *p*-value.

In the DL, AEX, and DK groups, 2097, 2475, and 2329 up-regulated DEGs were identified, respectively. Additionally, 2823, 3150, and 2771 DEGs were found to be down-regulated in the DL, AEX, and DK groups, respectively (Figure 3B). In all three ecotypes, the number of down-regulated DEGs exceeded that of the up-regulated DEGs. The Venn diagram illustrates that among the 2676 genes shared by the three ecotypes (Figure 3C), 1125 were up-regulated (Figure 3D), and 1478 were down-regulated (Figure 3E). Additionally, there were 1039, 1334, and 794 unique DEGs identified in DL, AEX, and DK, respectively (Figure 3C). The KEGG enrichment bubble plot (Figure 4A) for the top 20 metabolic pathways of the common up-regulated 1125 DEGs showed that the transcription of flavonoid biosynthesis was highly sensitive to low-temperature stress in sandrice. This result was consistent with the KEGG enrichment analysis of the up-regulated DEGs in the different comparison groups (Figure S2). In addition, the KEGG enrichment bubble plot (Figure 4B) of the common down-regulated 1478 DEGs included pathways, such as starch and sucrose metabolism (ko00500), glyoxylate and dicarboxylate metabolism (ko00630), pentose and glucuronate interconversions (ko00040), photosynthesis (ko00195), and photosynthesis-antenna proteins (ko00196). These findings suggested that sandrice responds to low-temperature stress by inhibiting most of the metabolic processes, such as photosynthesis, and sugar metabolism.

**Figure 5:**
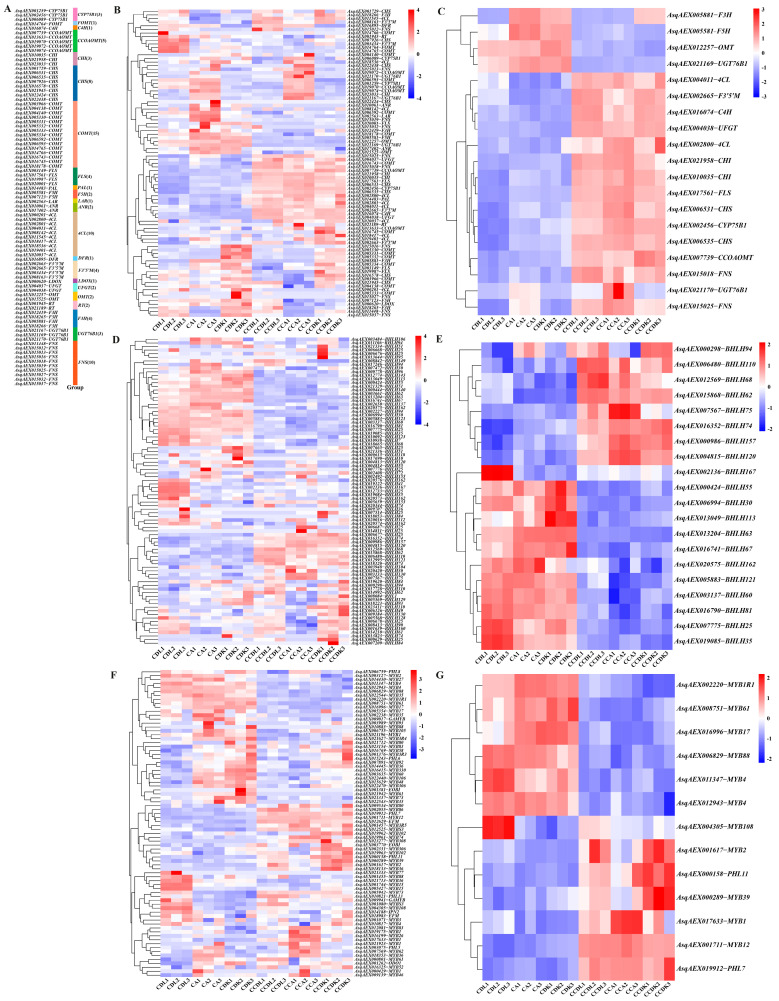
The hierarchical cluster heatmap of structure genes and TFs related to flavonoid synthesis in sandrice. (**A**) Classification of 85 flavonoid synthesis-related genes; (**B**) 85 genes related to flavonoid synthesis; (**C**) 19 common structure genes related to flavonoid synthesis; (**D**) 85 *bHLHs*; (**E**) 20 common differences in *bHLHs*; (**F**) 78 *MYBs*; (**G**) 13 common differences in *MYBs*.

Based on the sequence alignments of candidate genes, a total of 22 kinds of genes putatively involved in flavonoid synthesis were identified in the transcriptome of sandrice (Figure 5A). These included fifteen *COMTs*, ten *FNSs*, ten *4CLs*, eight *CHSs*, five *CCOAOMTs*, and other low copy genes, accounting for a total of eighty-five genes (Figure 5B). Also identified as potentially regulating flavonoid biosynthesis were 85 *bHLHs* (Figure 5C) and 78 *MYBs* (Figure 5D) TFs. Among these, there were 19 common DEGs related to flavonoid synthesis (Figure 5E), 20 common DEGs of *bHLHs* (Figure 5F), and 13 common DEGs of *MYBs* (Figure 5G). Interestingly, 15 out of 19 DEGs were up-regulated, which suggested the flavonoid synthesis pathway was positively involved in the adaptation of sandrice to cold stress. In contrast to the structural genes of flavonoid synthesis, the regulation of TFs showed a different pattern: among the 20 *bHLHs* analyzed, eleven were down-regulated and seven were up-regulated, while among the thirteen *MYB*s, six were down-regulated and six were up-regulated. This indicates a more complex regulatory behavior in these TFs when compared to the structural genes.

### 3.4. Integrative Analysis of DAFs and DEGs

**Figure 6:**
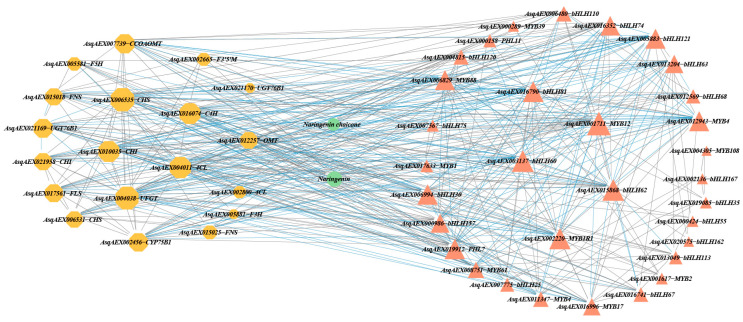
Correlation network diagram of DEGs and DAFs shared by the three ecotypes. Blue lines indicate a negative correlation between DEGs and DAFs, while gray lines indicate a positive correlation. Octagons, triangles, and circles are used to represent structural genes, TFs, and flavonoid metabolites, respectively.

To further explore the relationship between DEGs and DAFs, a correlation analysis was conducted on the common DEGs and DAFs among the three ecotypes. The correlation network between DEGs and DAFs revealed that there were nine core DEGs involved in regulating naringenin and naringenin chalcone synthesis, including *AsqAEX006535-CHS*, *AsqAEX016074*−*C4H*, *AsqAEX010035*−*CHI*, *AsqAEX004011*−*4CL*, *AsqAEX016790*−*bHLH81*, *AsqAEX001711*−*MYB12*, *AsqAEX015868*−*bHLH60*, *AsqAEX015868*−*bHLH62*, and *AsqAEX002220*−*MYB1R1* (Figure 6). Among them, *AsqAEX002220*−*MYB1R1* markedly negatively regulated the synthesis of both naringenin and naringenin chalcone, while *AsqAEX006535*−*CHS*, *AsqAEX016074*−*C4H*, *AsqAEX004011*−*4CL*, *AsqAEX001711*−*MYB12*, and *AsqAEX015868*−*bHLH62* markedly positively regulated the synthesis of naringenin (Table S5). Furthermore, *AsqAEX002220*−*MYB1R1*, *AsqAEX015868*−*bHLH62*, and *AsqAEX001711*−*MYB12* were identified as key TFs that regulated *AsqAEX006535*−*CHS*, *AsqAEX016074*−*C4H*, and *AsqAEX004011*−*4CL* to promote the accumulation of naringenin. Among these, *AsqAEX015868*−*bHLH62* and *AsqAEX001711*−*MYB12* were positively associated with *AsqAEX006535*−*CHS*, *AsqAEX016074*−*C4H*, and *AsqAEX004011*−*4CL*, whereas *AsqAEX002220*−*MYB1R1* was significantly negatively correlated with these structural genes.

**Figure 7:**
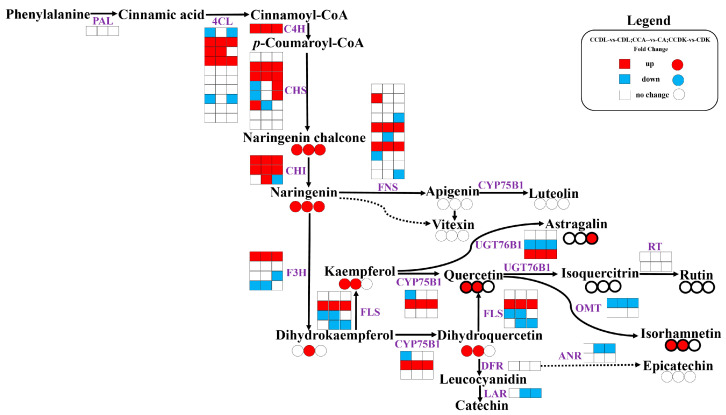
Flavonoid biosynthesis pathway in response to low-temperature stress in sandrice. The square and circle represent enzyme and flavonoid metabolite, respectively, and the three adjacent columns represent the three different ecotypes: DL, AEX, and DK. Red indicates that enzymes or flavonoid metabolites of the cold-treatment group are up-regulated compared to the control group, blue indicates that those are down-regulated, and white indicates that those had no significant change. Bolded black circles indicate more abundant metabolites. PAL, phenylalanine ammonia lyase; 4CL, 4-coumarate-CoA ligase; C4H, Cinnamate-4-hydroxylase; CHS, Chalcone synthase; CHI, Chalcone-flavanone isomerase; FNS, flavone synthase; CYP75B1, Flavonoid 3′-monooxygenase; F3H, Flavanone 3-dioxygenase; FLS, Flavanol synthase; UGT76B1, UDP-glycosyltransferase 76B1; OMT, O-methyltransferase; DFR, Dihydroflavonol-4-reductase; ANR, Anthocyanidin reductase; LAR, Leucoanthocyanidin reductase; and RT, Anthocyanidin-3-O-glucoside rhamnosyltransferase.

The flavonoid biosynthesis pathway in sandrice encompassing the 14 compounds detected was constructed according to the DEGs and DAFs (Figure 7). The pathway of sandrice flavonoid biosynthesis initiated with the transformation of phenylalanine to p-coumaroyl-CoA. Subsequently, a series of the enzymes were induced by cold stress to synthesize various stable flavonoids. These enzymes included five out of ten copies of 4CL, one C4H, five out of eight CHSs, three CHIs, seven out of ten FNSs, three out of four F3Hs, three out of four FLSs, two out of three CYP75B1s, two out of three UGT76B1s, one out of two OMTs, one out of two ANRs, and one LAR. The upstream pathways of flavonoid synthesis remained consistent across the three ecotypes, whereas the downstream pathways exhibited variability.

### 3.5. Q-PCR Validation of the Gene Expression Levels Related to Flavonoid Biosynthesis

**Figure 8:**
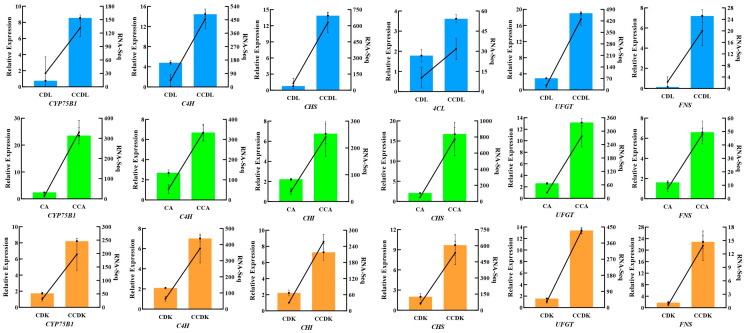
Q-PCR validation of the transcriptome data. The line graph and the histogram with error bar (±SE, *n* = 3) depict the relative expression calculated using the 2^−ΔΔCt^ method and the FPKM values generated by the RNA-Seq. The blue, green, and orange histograms represent genes examined in DL, AEX, and DK, respectively. *CYP75B1*, Flavonoid 3′-monooxygenase; *C4H*, Cinnamate-4-hydroxylase; *CHI*, Chalcone-flavanone isomerase; *CHS*, Chalcone synthase; *4CL*, 4-coumarate-CoA ligase; *UFGT*, Anthocyanidin 3-O-glucosyltransferase; and *FNS*, flavone synthase.

To further confirm the reliability and repeatability of the transcriptome data, this study conducted a Q-PCR analysis on the seven structural genes involved in the biosynthetic pathway of flavonoids. The findings revealed a consistent relative expression of these genes with their corresponding FPKM values obtained from the transcriptomic data (Figure 8).

## 4. Discussion

Sandrice is an important desert medicinal plant [36,37,38], and is widely distributed across various temperature zones [43]. Common garden experiments have demonstrated that the content of flavonoids varied among different ecotypes of sandrice, which was associated with habitat temperature heterogeneity [36,38]. To further elucidate the molecular metabolic regulatory mechanisms of flavonoid synthesis in response to low temperatures in sandrice, this study conducted ambient 4 °C low-temperature treatments, and compared the physiological, metabolic, and transcriptomic profiles. This research aims to provide a theoretical foundation for understanding the adaptability of desert plants to cold stress, and supports the precise utilization and development of the medicinal value of sandrice.

### 4.1. Low-Temperature Stress Impacts on the Photosynthesis of Sandrice

The morphological and phenotypic results indicated that the AGB, BBL, Plant Height, SD, and Leaf RWC of sandrice seedlings were barely affected by a four-day exposure to 4 °C cold stress. A study on cold stress in quinoa seedlings, a food crop native to the alpine regions of South America known for inheriting elite genes for cold-stress adaptation, found that a cold-tolerant quinoa line exhibited limited changes in Plant Height after 11 h of 5 °C low-temperature stress [44]. Similarly, sandrice also displayed a comparable cold tolerance to *F. tataricum,* a plant known for its robust resistance to cold environments [27]. These results suggested that sandrice exhibits strong tolerance to cold stress.

Additionally, photosynthesis, a fundamental process in plant physiology, serves as a crucial indicator for assessing the impact of abiotic stresses on plants [45]. This study observed significant changes in several representative photosynthesis parameters (Fv’/Fm’, Phi2, PhiNO, and PhiNPQ) in sandrice following low-temperature stress (Figure 1). Specifically, Fv’/Fm’ showed a significant reduction under low-temperature stress, indicating impaired maximum photosynthetic efficiency in the low-temperature treatment groups. Phi2 and PhiNO also markedly decreased in the cold-stressed group compared to the control, suggesting a reduced efficiency in light energy captured by PSII under cold stress. In contrast, PhiNPQ significantly increased in the cold-stressed group, indicating enhanced non-photochemical quenching and heightened photoprotection mechanisms in sandrice that mitigate damage. This result was consistent with previous studies that showed a weakened efficiency in converting light energy into chemical energy, and higher PhiNPQ levels as a protective mechanism for the photosynthetic system of leaves under stress [46,47]. Overall, while cold stress negatively impacts photosynthetic efficiency and light energy utilization in sandrice, the plant activates robust photoprotective mechanisms to manage the low-temperature stress effectively.

### 4.2. Flavonoid Biosynthesis Up-Regulated by Low-Temperature Stress in Sandrice

A total of 14 flavonoids were detected in sandrice (Table S2), with 12 of them showing an increased number of flavonoids after exposure to low-temperature stress. (Figure 2A). This observation indicated that sandrice responded to low-temperature stress by accumulating a large number of flavonoids. This phenomenon was supported by extensive research that indicated low-temperature stress can induce the accumulation of flavonoids in plants [24,25,27,48]. It was also found that five flavonoids, namely rutin, isoquercetin, astragalin, quercetin, and isorhamnetin, displayed relatively high content in sandrice, collectively accounting for over 99% of the total flavonoid content. These flavonoids showed increased accumulation under cold stress. When subjected to external stressors, plants can accumulate substantial flavonoids and other substances to mitigate the damage caused by the reactive oxygen species [49,50,51]. In addition, these flavonoid metabolites are known to possess various medicinal values, including antioxidant, hypoglycemic, antiviral, anti-inflammatory, anti-tumor, cardiovascular, and cerebrovascular protective properties [52,53,54,55,56,57]. This discovery underscores the rich flavonoid content in the above-ground tissue of sandrice, highlighting its potential as a valuable medicinal resource for development and application. Furthermore, in practical production, sandrice can be harvested after undergoing appropriate cold stress to ensure a high flavonoid content.

To screen the elite genetic resource for flavonoids industry, the molecular regulation mechanism of the flavonoid biosynthesis pathway responding to cold stress was explored through a joint analysis of DAFs and DEGs. The common DAFs among the three ecotypes were naringenin and naringenin chalcone under low-temperature stress, which serve as precursor substances for the other 12 flavonoid metabolites (Figure 7). A correlation network analysis revealed that cold stress significantly up-regulated at least three upstream DEGs, including *AsqAEX006535*−*CHS*, *AsqAEX016074*−*C4H*, and *AsqAEX004011*−*4CL*. These genes were positively regulated by *AsqAEX015868*−*bHLH62* and *AsqAEX001711*−*MYB12*, while also being negatively regulated by *AsqAEX002220*−*MYB1R1,* promoting the biosynthesis of naringenin in sandrice (Figure 6 and Table S5). Interestingly, *MYBs* and *bHLHs,* known to regulate flavonoid synthesis, are widely conserved across various plants species [38,58,59,60,61]. For example, in *L. barbarum LrMYB1* activates *LbCHS* to accumulate flavonoids [62,63], while some other MYBs, such as two R2R3-MYB proteins of poplar [64] and GbMYBF2 of *Ginkgo biloba* [65], inhibit flavonoid synthesis. In *Erigeron breviscapus*, *EbbHLH80* regulates the expression of some of the structural genes involved in flavonoid biosynthesis, influencing both flavonoid accumulation and plant development [66]. In this study, we identified both positive and negative regulators of flavonoid synthesis in response to cold stress in sandrice. However, further functional analyses are necessary to fully elucidate these mechanisms.

### 4.3. The Contradiction between Phytochemical Diversity and Genetic Diversity

Previous study has shown significant differences in phytochemical diversity among different ecotypes of *Rubia tinctorum*, which were greatly influenced by environmental factors [67]. In the present study, phytochemical diversity in flavonoid biosynthesis pathways was observed among different ecotypes of sandrice under cold stress. Specifically, DL and AEX exhibited similar accumulation patterns of flavonoid metabolites in response to cold stress, such as the up-regulation of kaempferol, quercetin, dihydroquercetin, and isorhamnetin in the downstream pathways. In contrast, DK showed a specific up-regulation of astragalin. These findings indicate that DL and AEX exhibit higher phytochemical diversity when compared to DK in response to cold stress, possibly due to their adaptation to high-altitude and high-latitude environments with lower temperatures. These findings aligned with a previous study that reported different flavonoid accumulation patterns in response to low-temperature stress among two *F. tataricum* landraces [27], potentially influenced by long-term domestication and evolutionary adaptation to local temperature conditions [68,69]. In addition, the diversification of flavonoid biosynthesis was also observed among different ecotypes of sandrice under drought stress (unpublished data).

On the other hand, previous population genetic analyses classified DK and DL within the *Central* lineage, while AEX was categorized into the *Eastern* lineage [43]. In this study, DL and DK also exhibited similar transcriptional responses to cold stress, distinct from AEX, which may indicate lineage-specific adaptations. Combined with the metabolism data, a conflict between genetic diversity (as evidenced by lineage distinctions) and phytochemical diversity (observed in flavonoid profiles) under cold stress was observed, highlighting the complex interplay between genetic regulation and environmental adaptation. Genetic lineage appears to influence broad transcriptional responses, while environmental factors such as cold stress can significantly alter metabolic pathways independent of genetic background. This underscores the necessity for integrated approaches combining genetics, transcriptomics, and metabolomics to comprehensively understand and manipulate plant responses to environmental stresses for agricultural and industrial purposes.

## 5. Conclusions

This study demonstrates that sandrice activates a robust photoprotection mechanism to dissipate excess energy under cold stress based on the physiological and transcriptome analyses. Metabolomic profiling further reveals that sandrice accumulates an abundance of flavonoids to defend against low temperatures. Furthermore, integrated analysis of common DEGs and DAFs unveils a unique flavonoid biosynthesis mechanism in sandrice under cold stress. Specifically, *AsqAEX006535*−*CHS*, *AsqAEX016074*−*C4H*, and *AsqAEX004011*−*4CL* could be fine-tuned by *AsqAEX015868*−*bHLH62*, *AsqAEX001711*−*MYB12,* and *AsqAEX002220*−*MYB1R1*, thereby regulating the accumulation of naringenin. Finally, based on the results of the flavonoid metabolisms, DL and AEX are suitable for the precise development of quercetin and isorhamnetin, while astragalin should be targeted for development in DK in the future. Although there are still many gaps to be filled, this study provides valuable preliminary data for elucidating the molecular metabolism of desert plants when adapting to cold stress, paving the way for precise utilization and development of the medicinal value of sandrice.

## Figures and Tables

**Table 1:** Environmental factors of the native habitats among the three ecotypes.

Ecotype Name	Lineage Name	Collection Site	Altitude Grouping	Altitude (m)	Latitude	Longitude	Mean Annual Precipitation (mm)	Annual Mean Temperature (°C)
DL	*Central*	Dulan	high altitude	3130	36°25′25.77″	98°7′25.35″	263	3.40
AEX	*Eastern*	Aerxiang	low altitude	251	42°52′4.80″	122°25′40.14″	485	6.38
DK	*Central*	Dengkou	middle altitude	1050	40°22′42.30″	106°59′37.51″	137	8.31

## Data Availability

The original contributions presented in the study are included in the article/Supplementary Material, further inquiries can be directed to the corresponding authors.

## Supplementary Materials

The following supporting information can be downloaded at: https://www.mdpi.com/article/10.3390/genes15091228/s1, Figure S1: The significant difference analysis of flavonoid-targeting metabolites in above-ground tissue of sandrice; Figure S2: KEGG enrichment analysis bubble map; Table S1: Primers used in Q-PCR; Table S2: Composition and content of flavonoid-targeted metabolites in sandrice; Table S3: DAFs in three different ecotypes of sandrice in response to low-temperature stress; Table S4: Summary of the RNA-Seq results of sandrice above-ground tissue; Table S5: PCC between DAFs and DEGs.
