# Supplementary material for: Flavonoid Synthesis Pathway Response to Low-Temperature Stress in a Desert Medicinal Plant, Agriophyllum Squarrosum (Sandrice)
Source: Genes (Basel). 2024 Sep 20;15(9):1228. doi: 10.3390/genes15091228 (PMC11431328; doi:10.3390/genes15091228)
Supplement: Supplementary file 1 [file genes-15-01228-s001.zip › Figure S1.pdf]

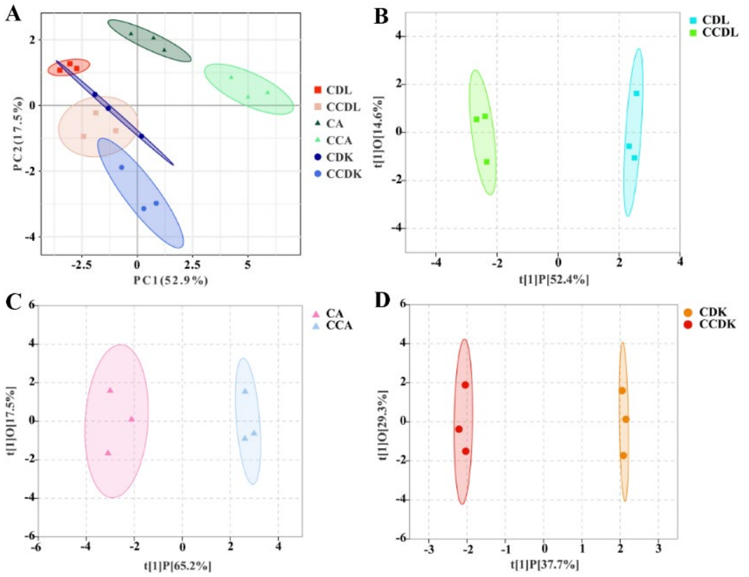

Figure S1. The significant difference analysis of flavonoid-targeting metabolites in above-ground tissue of sandrice. A, The PCA plot; B-D, Score scatter plot of OPLS-DA model for DL, AEX, and DK. PC1 and PC2 in the PCA analysis diagram represent the first principal component and the second principal component, respectively. In the scatter plot of OPLS-DA, the horizontal coordinate t[1]P represents the predicted principal component score of the first principal component, showing the differences between sample groups, and the vertical coordinate t[1]O represents the orthogonal principal component score, showing the differences within sample groups. Each scatter represents a sample, and the scatter shape and color represent different experimental groups. The percentage value in the figure represents the interpretation rate, and the larger the interpretation rate, the greater the sample difference. The ellipses in the figure represent 95% confidence intervals.
