# Supplementary material for: Flavonoid Synthesis Pathway Response to Low-Temperature Stress in a Desert Medicinal Plant, Agriophyllum Squarrosum (Sandrice)
Source: Genes (Basel). 2024 Sep 20;15(9):1228. doi: 10.3390/genes15091228 (PMC11431328; doi:10.3390/genes15091228)
Supplement: Supplementary file 1 [file genes-15-01228-s001.zip › Figure S2.pdf]

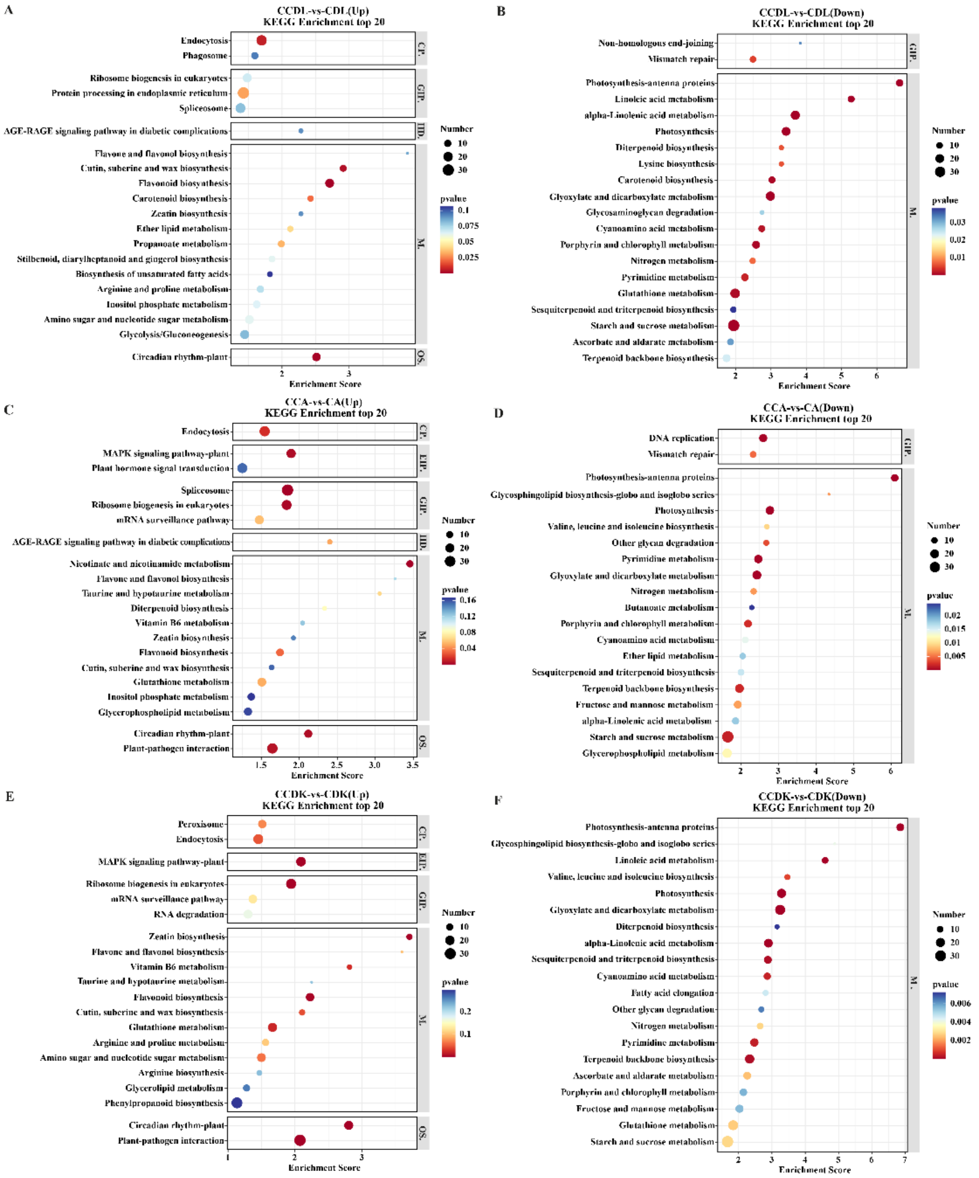

Figure S2. KEGG enrichment analysis bubble map. A, The bubble map of up-regulated DEGs in DL; B, The bubble map of down-regulated DEGs in DL; C, The bubble map of up-regulated DEGs in AEX; D, The bubble map of down-regulated DEGs in AEX; E, The bubble map of up-regulated DEGs in DK; F, The bubble map of down-regulated DEGs in DK; CP., GIP., M., OS., EIP, and HD represents Cellular Processes, Genetic Information Processing, Metabolism, Organismal Systems, Environmental Information Processing, and Human Diseases, respectively. Dot size represents the number of distinct genes, and dot color reflects the *p*-value.
