# Supplementary material for: Flavonoid Synthesis Pathway Response to Low-Temperature Stress in a Desert Medicinal Plant, Agriophyllum Squarrosum (Sandrice)
Source: Genes (Basel). 2024 Sep 20;15(9):1228. doi: 10.3390/genes15091228 (PMC11431328; doi:10.3390/genes15091228)
Supplement: Supplementary file 1 [file genes-15-01228-s001.zip › Table S1.pdf]

Table S1. Primers used in Q-PCR.

Gene Name	Gene No.	Forward primer (5' to 3')	Reverse primer (5' to 3')	Primer TM value	Product length
UBC22	*AsqAEX016065*	AATGGAGCCCAGCACTTACA	GCCTCATACTTTGGCCGATCA	57	133
PP2A	*AsqAEX007496*	TCGCCCCTGTTTAGTGGAGT	GTACGTCTTCTCTGCCGACT	58	122
CYP75B1	*AsqAEX002456*	TGGGATGAGTTTGGGCCTTC	CTTTCGTTGCAAGGTCAGCC	60	139
C4H	*AsqAEX016074*	CCACCCAGAGATCCAGAGGA	AATGGCCATACGCAACCTCA	60	142
CHI	*AsqAEX010035*	TCTGGAAGGCCATCGGAGTA	AAAATAGAGTGGCCCGGTGG	60	103
CHS	*AsqAEX006535*	TGAATCCATTGAGCGGCCAT	TCAGGCCTACTTCACGCAAG	60	104
4CL	*AsqAEX004011*	GGGCTATGGAATGACGGAGG	GGCACCAGTGTCAGGATCAA	60	142
UFGT	*AsqAEX004038*	GTGTCATGGGCACCACAAAC	AATGGCCTTCCGATCAGTGG	60	125
FNS	*AsqAEX015025*	CAAGCCCGTCCTTGTGGTAT	GCCACATCCCTACTGCCATT	60	129
