# Supplementary material for: Flavonoid Synthesis Pathway Response to Low-Temperature Stress in a Desert Medicinal Plant, Agriophyllum Squarrosum (Sandrice)
Source: Genes (Basel). 2024 Sep 20;15(9):1228. doi: 10.3390/genes15091228 (PMC11431328; doi:10.3390/genes15091228)
Supplement: Supplementary file 1 [file genes-15-01228-s001.zip › Table S2.pdf]

Table S2. Composition and content of flavonoid-targeted metabolites in sandrice after low-temperature stress (ng/100mg).

	Content (ng/100mg, FW)					
**Compound name**	**CDL**	**CCDL**	**CA**	**CCA**	**CDK**	**CCDK**
Rutin	3750.754	3901.067	14998.187	18284.928	3785.641	4464.343
Isoquercitrin	7263.922	8979.420	9694.873	10851.357	10863.620	11081.077
Astragalin	1959.514	2450.355	3770.249	4457.543	1563.669	2107.902
Quercetin	209.529	367.249	1377.841	3031.893	650.892	1097.557
Isorhamnetin	493.321	696.573	979.875	1473.388	1045.934	2022.420
Kaempferol	9.332	27.044	87.774	214.162	20.997	38.279
Dihydroquercetin	11.369	16.450	20.696	47.264	24.431	35.263
Vitexin	30.018	20.054	16.188	12.777	16.202	17.760
Dihydrokaempferol	5.519	7.221	12.121	29.501	4.457	4.973
Luteolin	1.131	1.362	2.195	3.888	1.946	1.990
Naringenin	1.978	5.509	0.737	3.630	1.487	3.828
Naringenin chalcone	1.254	2.845	0.733	3.024	1.796	4.898
Apigenin	0.245	0.344	0.445	1.065	0.547	0.391
Epicatechin	0.701	0.978	0.265	0.500	0.049	0.297
total	13738.587	16476.471	30962.179	38414.920	17981.668	20880.978
