# Supplementary material for: Flavonoid Synthesis Pathway Response to Low-Temperature Stress in a Desert Medicinal Plant, Agriophyllum Squarrosum (Sandrice)
Source: Genes (Basel). 2024 Sep 20;15(9):1228. doi: 10.3390/genes15091228 (PMC11431328; doi:10.3390/genes15091228)
Supplement: Supplementary file 1 [file genes-15-01228-s001.zip › Table S3.pdf]

Table S3. Screening of DAFs in three sandrice ecotypes after low-temperature stress.

Compound name	CCDL-vs-CDL			CCA-vs-CA			CCDK-vs-CDK		
	**Fold Change**	**VIP**	***p*-value**	**Fold Change**	**VIP**	***p*-value**	**Fold Change**	**VIP**	***p*-value**
Rutin	1.04	0.11	0.84	1.22	0.95	0.07	1.18	1.03	0.19
Isoquercitrin	1.24	1.12	0.06	1.12	0.60	0.36	1.02	0.08	0.88
**Astragalin**	1.25	0.51	0.41	1.18	0.81	0.16	**1.35**	**1.34**	**0.03**
**Quercetin**	**1.75**	**1.12**	**0.01**	**2.20**	**1.11**	**0.01**	1.69	0.84	0.26
**Isorhamnetin**	**1.41**	**1.03**	**0.05**	**1.50**	**1.04**	**0.04**	1.93	1.25	0.07
**Kaempferol**	**2.90**	**1.29**	**0.00**	**2.44**	**1.14**	**0.00**	1.82	1.03	0.16
**Dihydroquercetin**	**1.45**	**1.25**	**0.04**	**2.28**	**1.20**	**0.00**	1.44	1.24	0.12
Vitexin	0.67	0.78	0.28	0.79	0.66	0.26	1.10	0.52	0.80
**Dihydrokaempferol**	1.31	0.68	0.33	**2.43**	**1.19**	**0.00**	1.12	0.39	0.61
Luteolin	1.20	1.08	0.11	1.77	1.06	0.06	1.02	0.01	0.92
**Naringenin**	**2.79**	**1.17**	**0.00**	**4.93**	**1.21**	**0.01**	**2.57**	**1.51**	**0.00**
**Naringenin Chalcone**	**2.27**	**1.23**	**0.03**	**4.13**	**1.20**	**0.00**	**2.73**	**1.43**	**0.00**
Apigenin	1.40	0.93	0.14	2.39	1.06	0.10	0.72	0.98	0.30
Epicatechin	1.39	0.96	0.13	1.89	0.07	0.67	6.01	0.77	0.42

Note: Screening criteria for flavonoid differential metabolites were VIP ≥ 1 and $p \leq 0.05$, and a total of 8 DAFs were selected.
