# Supplementary material for: Flavonoid Synthesis Pathway Response to Low-Temperature Stress in a Desert Medicinal Plant, Agriophyllum Squarrosum (Sandrice)
Source: Genes (Basel). 2024 Sep 20;15(9):1228. doi: 10.3390/genes15091228 (PMC11431328; doi:10.3390/genes15091228)
Supplement: Supplementary file 1 [file genes-15-01228-s001.zip › Table S4.pdf]

Table S4. Summary of the RNA-Seq results of sandrice aboveground tissue.

Sample	Raw Data (G)	Clean Data (G)	Q30 (%)	Uniquely mapped	Total mapped reads	GC (%)
CDL1	7.11	6.76	92.82	93.64%	96.62%	43.79
CDL2	6.61	6.29	92.68	93.63%	96.58%	43.86
CDL3	6.63	6.35	92.71	93.61%	96.57%	43.84
CCDL1	7.24	6.92	92.71	91.84%	96.57%	43.55
CCDL2	6.58	6.29	92.63	92.04%	96.40%	43.38
CCDL3	7.07	6.77	92.8	92.21%	96.59%	43.43
CA1	7.17	6.87	92.81	95.26%	98.39%	43.92
CA2	7.32	6.99	92.96	95.28%	98.47%	44.23
CA3	7.11	6.79	92.81	95.24%	98.36%	44.21
CCA1	7.3	6.98	93.17	93.52%	98.57%	43.55
CCA2	7.24	6.92	93.17	93.79%	98.53%	43.64
CCA3	7.22	6.93	92.87	93.53%	98.42%	43.66
CDK1	6.68	6.4	92.86	91.09%	96.80%	44.43
CDK2	7.24	6.88	92.77	90.53%	96.72%	44.49
CDK3	7.24	6.96	92.65	93.93%	96.89%	44.29
CCDK1	7.09	6.77	92.9	91.41%	96.71%	43.58
CCDK2	7.17	6.86	92.95	91.63%	96.70%	43.48
CCDK3	7.16	6.87	92.85	92.60%	96.82%	43.56
