# Supplementary material for: Flavonoid Synthesis Pathway Response to Low-Temperature Stress in a Desert Medicinal Plant, Agriophyllum Squarrosum (Sandrice)
Source: Genes (Basel). 2024 Sep 20;15(9):1228. doi: 10.3390/genes15091228 (PMC11431328; doi:10.3390/genes15091228)
Supplement: Supplementary file 1 [file genes-15-01228-s001.zip › Table S5.pdf]

Table S5. PCC between DAFs and DEGs.

**node1**	**node2**	**PCC**	***p*-value**
AsqAEX006535-CHS	Naringenin	0.818139	0.000033
AsqAEX016074-C4H	Naringenin	0.917688	0.000000
AsqAEX004011-4CL	Naringenin	0.85407	0.000006
AsqAEX015868-bHLH62	Naringenin	0.935859	0.000000
AsqAEX002220-MYB1R1	Naringenin	-0.85382	0.000007
AsqAEX002220-MYB1R1	Naringenin chalcone	-0.81223	0.000042
AsqAEX001711-MYB12	Naringenin	0.82829	0.000022
AsqAEX006535-CHS	*AsqAEX015868-bHLH62*	0.896456	0.000000
AsqAEX006535-CHS	*AsqAEX002220-MYB1R1*	-0.86043	0.000005
AsqAEX006535-CHS	*AsqAEX001711-MYB12*	0.977907	0.000000
AsqAEX016074-C4H	*AsqAEX015868-bHLH62*	0.969707	0.000000
AsqAEX016074-C4H	*AsqAEX002220-MYB1R1*	-0.87967	0.000002
AsqAEX016074-C4H	*AsqAEX001711-MYB12*	0.879813	0.000001
AsqAEX004011-4CL	*AsqAEX015868-bHLH62*	0.923681	0.000000
AsqAEX004011-4CL	*AsqAEX002220-MYB1R1*	-0.90636	0.000000
AsqAEX004011-4CL	*AsqAEX001711-MYB12*	0.905168	0.000000
